# Chest radiography versus lung ultrasound for identification of acute respiratory distress syndrome: a retrospective observational study

**DOI:** 10.1186/s13054-018-2105-y

**Published:** 2018-08-18

**Authors:** Kay Choong See, Venetia Ong, Yi Lin Tan, Juliet Sahagun, Juvel Taculod

**Affiliations:** 1 Division of Respiratory & Critical Care Medicine, University Medicine Cluster, National University Health System, 1E Kent Ridge Road, NUHS Tower Block Level 10, Singapore, 119228 Singapore; 2 Department of Medicine, Yong Loo Lin School of Medicine, National University of Singapore, Singapore, Singapore; 3 Department of Medical Affairs, National University Hospital, Singapore, Singapore; 4 Yong Loo Lin School of Medicine, National University of Singapore, Singapore, Singapore; 5 Division of Critical Care—Respiratory Therapy, National University Hospital, Singapore, Singapore

**Keywords:** Diagnosis, Lung, Radiography, Radiology, Respiratory distress syndrome, adult, Ultrasonography

## Abstract

**Background:**

Lung ultrasound may be a reasonable alternative to chest radiography for the identification of acute respiratory distress syndrome (ARDS), but the diagnostic performance of lung ultrasound for ARDS is uncertain. We therefore analyzed the clinical outcomes of ARDS diagnosed according to the Berlin Definition, using either chest radiography (Berlin-CXR) or lung ultrasound (Berlin-LUS) as an alternative imaging method.

**Methods:**

This was a retrospective observational study in a 20-bed medical intensive care unit (ICU). Patients who required noninvasive ventilation or invasive ventilation for hypoxemic respiratory failure on ICU admission from August 2014 to March 2017 were included. Both chest radiography and lung ultrasound were performed routinely upon ICU admission. Comparisons were made using either the Berlin-CXR or Berlin-LUS definitions to diagnose ARDS with respect to the patient characteristics and clinical outcomes for each definition. ICU and hospital mortality were the main outcome measures for both definitions.

**Results:**

The first admissions of 456 distinct patients were analyzed. Compared with the 216 patients who met the Berlin-CXR definition (ICU mortality 19.4%, hospital mortality 36.1%), 229 patients who met the Berlin-LUS definition (ICU mortality 22.7%, hospital mortality 34.5%) and 79 patients who met the Berlin-LUS but not the Berlin-CXR definition (ICU mortality 21.5%, hospital mortality 29.1%) had similar outcomes. In contrast, the 295 patients who met either definition had higher mortality than the 161 patients who did not meet either definition (ICU mortality 20.0% versus 12.4%, *P* = 0.041; hospital mortality 34.2% versus 24.2%, *P* = 0.027). Compared with Berlin-CXR, Berlin-LUS had a positive predictive value of 0.66 (95% confidence interval 0.59–0.72) and a negative predictive value of 0.71 (0.65–0.77). Among the 216 Berlin-CXR ARDS patients, 150 patients (69.4%) also fulfilled Berlin-LUS definition.

**Conclusions:**

For the identification of ARDS using the Berlin definition, both chest radiography and lung ultrasound were equally related to mortality. The Berlin definition using lung ultrasound helped identify patients at higher risk of death, even if these patients did not fulfill the conventional Berlin definition using chest radiography. However, the moderate overlap of patients when chest imaging modalities differed suggests that chest radiography and lung ultrasound should be complementary rather than used interchangeably.

**Electronic supplementary material:**

The online version of this article (10.1186/s13054-018-2105-y) contains supplementary material, which is available to authorized users.

## Background

Acute respiratory distress syndrome (ARDS) has high mortality and requires specialized therapy. The current diagnostic criteria for ARDS follows the Berlin definition, which requires chest radiography or computed tomography for determination of bilateral pulmonary infiltrates [1]. Alternatively, the Kigali Modification of the Berlin definition has been proposed which, among other changes, allows for either chest radiography or lung ultrasound to be used for the determination of bilateral pulmonary infiltrates [2].

Chest radiography has certain drawbacks. Firstly, chest radiography has poor sensitivity for the detection of pulmonary infiltrates compared with other imaging modalities such as computed tomography [3]. Secondly, chest radiography has poor specificity for the determination of acute lung injury [4]. Thirdly, chest radiography may not be available in resource-limited settings, or in austere environments, and requires the use of radiation [5].

Lung ultrasound may be a useful alternative to chest radiography for the identification of ARDS. It has been established as an important tool for the evaluation of critically ill patients, with higher sensitivity and specificity than chest radiography for the diagnosis of pneumonia, using computed tomography as the reference standard [6, 7]. It has also been shown to be useful in diagnosing specific pathological features such as interstitial edema, consolidation, and pleural effusion [4, 8, 9]. Using transpulmonary thermodilution measurement as the reference standard for pulmonary edema, lung ultrasound predicted extravascular lung water in ventilated intensive care patients better than chest radiography [10]. Finally, low-cost, highly portable systems are now available for use in resource-limited settings [5].

Apart from a small pilot study [11] and a recent evaluation of the Kigali Modification [12], the use of chest radiography versus the use of lung ultrasound for the identification of ARDS has not been systematically studied. It is also unclear how the choice of imaging would affect ARDS identification. One common way to determine the validity of any set of disease criteria would be to assess its association with clinically important outcomes; for ARDS, intensive care unit (ICU) and hospital mortality would be the main outcome measures [1, 13, 14]. Therefore, we aimed to analyze the clinical outcomes of ARDS diagnosed according to the Berlin definition using either chest radiography or modified using lung ultrasound. Our hypothesis is that lung ultrasound is as good as, or better than, chest radiography at defining patients with ARDS, thereby providing more options for the early identification of ARDS in both resource-rich and resource-poor settings. Additionally, we aimed to evaluate the diagnostic accuracy of various lung ultrasound-based definitions against the Berlin definition using chest radiography.

## Methods

### Study design and patients

This study involved a retrospective analysis of patients who required noninvasive ventilation or invasive ventilation for hypoxemic respiratory failure (i.e., pulse oximetry-derived peripheral capillary oxygen saturation < 90% on room air), on admission to our 20-bed medical ICU within our 1200-bed tertiary hospital from August 2014 to March 2017 (32 months). Only the first admissions to ICU during the study period for patients whose diagnosis of respiratory failure was within 1 week of a known clinical insult or new/worsening respiratory symptoms, not fully explained by heart failure or fluid overload, were included for analysis. Chest radiography, lung ultrasound, and arterial blood gas measurements were performed routinely upon ICU admission, all within 60 min, and on the same ventilator settings. Ethical approval for the study was obtained from the National Healthcare Group Domain-Specific Review Board (DSRB reference 2013/00132). The need for informed consent was waived.

### Data collection

Data were obtained from the hospital case notes and ICU electronic patient records (IntelliSpace Critical Care and Anesthesia information system, Philips Singapore). Patient demographics, main diagnoses from the ICU admission notes, comorbid conditions, the source of ICU admission (general ward versus emergency department), inspired oxygen fraction, arterial blood gases, chest radiography results, and lung ultrasound results were collected at the time of ICU admission. Patients on noninvasive ventilation had positive end-expiratory pressure values recorded at the time of ICU admission. Patients on invasive mechanical ventilation had plateau pressures and positive end-expiratory pressure values recorded at the time of ICU admission. Clinical outcome data collected included ventilator-free days through 28 days, ICU length-of-stay, hospital length-of-stay, ICU mortality, and hospital mortality.

### Defining ARDS

ARDS was defined in two ways: using a combination of the Berlin definition with chest radiography (Berlin-CXR) or modified using lung ultrasound as an alternative imaging method (Berlin-LUS). For both definitions, diagnosis of respiratory failure was within 1 week of a known clinical insult or new/worsening respiratory symptoms, not fully explained by heart failure or fluid overload. For both ARDS definitions, patients on noninvasive ventilation could be classified as having mild ARDS but not moderate or severe ARDS [1, 9]. With regards to oxygenation, patients with ARDS required the ratio of arterial partial pressure of oxygen to inspired fraction of oxygen to be 300 mmHg or lower. For the Berlin-CXR definition, the imaging criterion required the presence of bilateral radiographic opacities not fully explained by pleural effusions, lobar/lung collapse, or nodules [14]. For the Berlin-LUS definition, the imaging criterion required at least one region of each hemithorax to be affected by multiple B lines (> 2 B lines per rib space) or consolidation on lung ultrasound. However, consolidation adjacent to pleural effusion could be a consequence of pulmonary atelectasis and was not counted as positive for ARDS.

Portable chest radiography was routinely performed at the bedside by the on-call radiographer, with images reported within a few hours by a radiologist who had no direct contact with patients. All radiological interpretations and confirmation of reports were performed by specialist radiologists in our hospital.

Lung ultrasound was also routinely performed at the bedside using the Sparq Ultrasound System (Philips Healthcare, Andover, MA) equipped with a 2–4 MHz broadband sector phased array transducer, and concurrently reported using a reporting template within the ICU electronic patient record by trained respiratory therapists (see Additional file 1: Table S1). The respiratory therapists underwent a standardized curriculum, completed at least 10 ultrasound scans during training, and passed a practical assessment [15]. All ultrasound images were interpreted immediately outside the patient’s room after image acquisition, based on cine images saved in the machine. Six regions of each hemithorax (anterior-superior, anterior-inferior, lateral-superior, lateral-inferior, posterior-superior, and posterior-inferior) were scanned for the presence of B lines, consolidation, and pleural effusion [16]. B lines were hyperechoic lines extending vertically from the pleural line to the opposite screen edge, indicating alveolar-interstitial edema. Consolidation could be demonstrated by hypoechoic or hyperechoic artifacts that disrupted the pleural line, or by sonographic air bronchograms. Within each region, having two or fewer B lines in the absence of consolidation was considered to be normal [15]. Consolidation adjacent to pleural effusion could be a consequence of pulmonary atelectasis and was thus not considered to represent pulmonary infiltrates [15].

### Statistical analysis

Univariate comparisons of proportions, means, and medians were respectively performed using the Pearson chi-squared, Student *t*, and Wilcoxon rank-sum tests. Statistical significance was taken as *P* < 0.05. A comparison was done using three definitions of ARDS (Berlin-CXR, Berlin-LUS, and combined Berlin-CXR or Berlin-LUS) with respect to patient characteristics, treatment parameters, and clinical outcomes. Imaging findings of patients who met the Berlin-CXR but not the Berlin-LUS definition, and vice versa, were described.

To analyze the appropriateness of using at least one region affected by multiple B lines (> 2 B lines per region) or consolidation to define ARDS, the lung ultrasound criterion for ARDS was increased progressively from at least one region to all six regions of each hemithorax affected by multiple B lines (> 2 B lines per region) or consolidation, and the clinical outcomes of patients identified to have ARDS were described. The diagnostic accuracy of various lung ultrasound-based definitions was then tested against Berlin-CXR as the reference standard for ARDS.

## Results

**Fig. 1:**
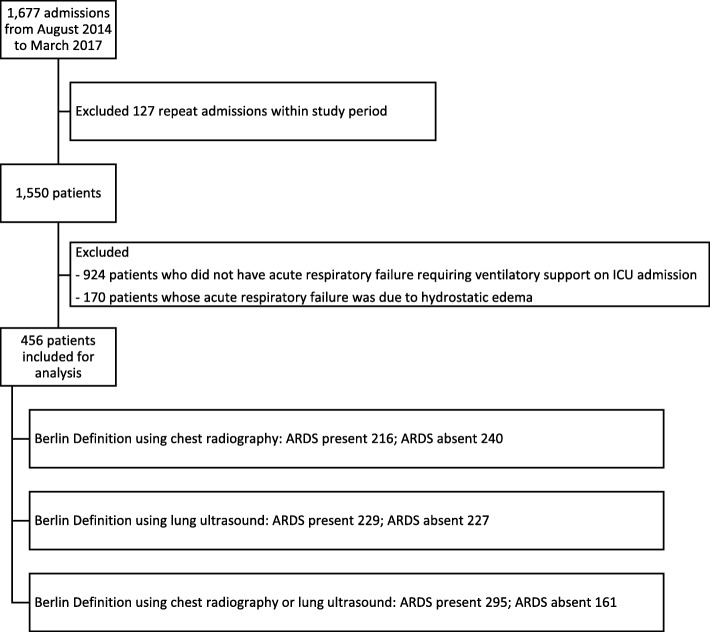
Study flowchart. ARDS acute respiratory distress syndrome, ICU intensive care unit

During the 32-month study period, out of 1677 ICU admissions of 1550 patients, the first admissions of 456 distinct patients were analyzed (Fig. 1). Using the Berlin definition and chest radiography (Berlin-CXR), 216 patients had ARDS. When modifying the Berlin definition to use lung ultrasound for chest imaging (Berlin-LUS), 229 patients had ARDS. Among the 216 Berlin-CXR ARDS patients, 150 patients (69.4%) also fulfilled Berlin-LUS definition. Among the 229 Berlin-LUS ARDS patients, 150 patients (65.5%) also fulfilled Berlin-CXR definition, and 295 patients fulfilled either the Berlin-CXR or the Berlin-LUS definition (Table 1).

**Table 1:** Patient characteristics

Characteristics	All patients	Berlin-CXR		Berlin-LUS		Berlin-CXR or Berlin-LUS	
		ARDS present	ARDS absent	ARDS present	ARDS absent	ARDS present	ARDS absent
*n*	456	216	240	229	227	295	161
Age (years)	62.3 ± 14.8	63.1 ± 14.3	61.5 ± 15.3	62.3 ± 14.3	62.2 ± 15.4	62.9 ± 14.3	61.2 ± 15.7
Female, *n* (%)	163 (35.8)	70 (32.4)	93 (38.8)	75 (32.8)	88 (38.8)	100 (33.9)	63 (39.1)
Height (m)	1.60 ± 0.09	1.60 ± 0.09	1.60 ± 0.09	1.60 ± 0.09	1.60 ± 0.10	1.60 ± 0.09	1.60 ± 0.10
Weight (kg)	64.7 ± 17.9	66.7 ± 18.8*	62.9 ± 16.8*	64.8 ± 16.8	64.6 ± 18.9	65.4 ± 17.9	63.3 ± 17.8
ED (vs non-ED) admission, *n* (%)	294 (64.5)	126 (58.3)*	168 (70.0)*	137 (59.8)*	157 (69.2)*	182 (61.7)	112 (69.6)
Main diagnosis, *n* (%)		*	*	*	*	*	*
Pneumonia	129 (28.3)	90 (41.7)	39 (16.3)	85 (37.1)	44 (19.4)	113 (38.3)	16 (9.9)
Other sepsis^a^	51 (11.2)	26 (12.0)	25 (10.4)	25 (10.9)	26 (11.5)	32 (10.9)	19 (11.8)
COPD/asthma	22 (4.8)	10 (4.6)	12 (5.0)	8 (3.5)	14 (6.2)	14 (4.8)	8 (5.0)
AMI	9 (2.0)	4 (1.9)	5 (2.1)	4 (1.8)	5 (2.2)	4 (1.4)	5 (3.1)
Stroke	51 (11.2)	14 (6.5)	37 (15.4)	18 (7.9)	33 (14.5)	23 (7.8)	28 (17.4)
Other^b^	194 (42.5)	72 (33.3)	122 (50.8)	89 (38.9)	105 (46.3)	109 (37.0)	85 (52.8)
Comorbidity, *n* (%)							
DM	175 (38.4)	86 (39.8)	89 (37.1)	81 (35.4)	94 (41.4)	109 (37.0)	66 (41.0)
Hypertension	258 (56.6)	122 (56.5)	136 (56.7)	124 (54.2)	134 (59.0)	165 (55.9)	93 (57.8)
IHD	89 (19.5)	47 (21.8)	42 (17.5)	54 (23.6)*	35 (15.4)*	65 (22.0)	24 (14.9)
CCF	10 (2.2)	6 (2.8)	4 (1.7)	6 (2.6)	4 (1.8)	8 (2.7)	2 (1.2)
COPD	18 (4.0)	10 (4.7)	8 (3.3)	8 (3.5)	10 (4.4)	12 (4.1)	6 (3.7)
CKD	92 (20.2)	49 (22.7)	43 (17.9)	50 (21.8)	42 (18.5)	64 (21.7)	28 (17.4)
CLD	42 (9.2)	22 (10.2)	20 (8.3)	21 (9.2)	21 (9.3)	27 (9.2)	15 (9.3)
Cancer	42 (9.2)	21 (9.7)	21 (8.9)	26 (11.4)	16 (7.1)	30 (10.2)	12 (7.5)
APACHE II	28.4 ± 8.0	29.5 ± 8.0*	27.5 ± 7.9*	28.9 ± 8.1	27.9 ± 7.9	29.1 ± 8.1*	27.2 ± 7.7*
pH	7.32 ± 0.15	7.32 ± 0.15	7.33 ± 0.15	7.33 ± 0.15	7.32 ± 0.14	7.32 ± 0.15	7.33 ± 0.15
PF ratio	218 ± 152	150 ± 70*	280 ± 177*	149 ± 66*	288 ± 180*	152 ± 67*	343 ± 184*

All values are shown as mean ± standard deviation unless otherwise indicated

*AMI* acute myocardial infarction, *APACHE* Acute Physiology and Chronic Health Evaluation score, *ARDS* acute respiratory distress syndrome, *CCF* Congestive cardiac failure, *COPD* chronic obstructive pulmonary disease, *CKD* chronic kidney disease, *CLD* chronic liver disease, *CXR* chest radiography, *DM* diabetes mellitus, *ED* Emergency department, *IHD* ischemic heart disease, *LUS* lung ultrasound, *PF ratio* partial pressure of oxygen in arterial blood divided by the inspired oxygen fraction

^a^For example, urinary tract infection, hepatobiliary sepsis

^b^For example, massive gastrointestinal bleeding, massive hemoptysis, pulmonary vasculitis, pneumonitis, pulmonary embolism, malignancy, endocrine emergencies, nonstroke neuromuscular emergencies

*P < 0.05; for main diagnosis, a 6 × 2 Pearson chi-squared test was performed for each ARDS definition

Out of 295 patients who met either the Berlin-CXR or Berlin-LUS definitions, 66 (22.4%) patients met the Berlin-CXR but not the Berlin-LUS definition (Table 2), while 79 (26.8%) patients met the Berlin-LUS but not the Berlin-CXR definition (Table 3). Among the 66 patients who met the Berlin-CXR definition for ARDS but not the Berlin-LUS definition, 61 (92.4%) had CXR opacities not detected by lung ultrasound, while 5 (7.6%) patients had apparent consolidation on CXR that was found to be pleural effusion. Conversely, among the 79 patients who met the Berlin-LUS definition for ARDS but not the Berlin-CXR definition, 68 (86.1%) had sonographic consolidation not detected by CXR, while 52 (65.8%) patients had multiple B lines (> 2 B lines per region) not detected by CXR. More B lines and consolidations were detected by ultrasound in the posterior and inferior regions, rather than in the anterior and superior regions.

**Table 2:** Imaging findings of patients who met Berlin-CXR but not Berlin-LUS definition

Imaging findings	Berlin-CXR, but not Berlin-LUS definition met (*n* = 66)
CXR opacity not detected by LUS on the right side^a^ (%)	45 (68.2)
CXR opacity not detected by LUS on the left side^a^ (%)	37 (56.1)
CXR opacity excluded due to pleural effusion detected by LUS on the right (%)	2 (3.0)
CXR opacity excluded due to pleural effusion detected by LUS on the left (%)	3 (4.5)

^a^Right side on chest radiography (CXR) was compared with any of the right-sided regions on lung ultrasound (LUS). Left side on CXR was compared with any of the left-sided regions on LUS; for example, if a right-sided opacity on CXR could be detected by the presence of either multiple B lines (> 2 B lines per region) or consolidation in any one of the right sided regions, it was deemed to be detected by both CXR and LUS, and if consolidation was detected in the left posterior-inferior region on LUS and if there was also a left-sided opacity on CXR, it was deemed to be detected by both CXR and LUS

**Table 3:** Imaging findings of patients who met Berlin-LUS but not Berlin-CXR definition

Imaging findings	Berlin-LUS, but not Berlin-CXR definition met (*n* = 79)
LUS multiple B lines (> 2 B lines per region) not detected by CXR^a^	
Anterior-superior (%)^b^	13 (16.5)
Anterior-inferior (%)^b^	15 (19.0)
Lateral-superior (%)^b^	18 (22.8)
Lateral-inferior (%)^b^	29 (36.7)
Posterior-superior (%)^b^	26 (32.9)
Posterior-inferior (%)^b^	29 (36.7)
LUS consolidation not detected by CXR^a^	
Anterior-superior (%)^b^	22 (27.8)
Anterior-inferior (%)^b^	27 (34.2)
Lateral-superior (%)^b^	30 (38.0)
Lateral-inferior (%)^b^	28 (35.4)
Posterior-superior (%)^b^	24 (30.4)
Posterior-inferior (%)^b^	35 (44.3)

^a^Right side on chest radiography (CXR) was compared with any of the right-sided regions on lung ultrasound (LUS). Left side on CXR was compared with any of the left-sided regions on LUS; for example, if a right-sided opacity on CXR could be detected by the presence of either multiple B lines (> 2 B lines per region) or consolidation in any one of the right sided regions, it was deemed to be detected by both CXR and LUS, and if consolidation was detected in the left posterior-inferior region on LUS and if there was also a left-sided opacity on CXR, it was deemed to be detected by both CXR and LUS

^b^Right and left sides combined; if either the corresponding right-sided region or left-sided region was detected on LUS but not CXR, it was deemed that the regions were detected by LUS but not detected by CXR

With regard to initial treatment parameters, those who had ARDS required more noninvasive ventilation support, higher initial positive end-expiratory pressure, and higher initial plateau pressure compared with patients who did not have ARDS (see Additional file 2: Table S2). Using Berlin-CXR, clinical outcomes of patients who had ARDS were worse than those who did not have ARDS: fewer 28-day ventilator-free days, greater ICU length-of-stay, and greater hospital mortality. Similarly, using Berlin-LUS, clinical outcomes of patients who had ARDS were worse than those who did not have ARDS: greater ICU length-of-stay and greater ICU mortality. Patients who met the Berlin-LUS definition for ARDS but not the Berlin-CXR definition had similarly poor outcomes compared with patients identified to have ARDS by Berlin-CXR (Tables 4 and 5). Compared with Berlin-CXR, Berlin-LUS had a positive predictive value of 0.66 (95% confidence interval 0.59–0.72) and a negative predictive value of 0.71 (0.65–0.77).

**Table 4:** Comparison of clinical outcomes of patients who met Berlin-CXR definition versus patients who met Berlin-LUS but not Berlin-CXR definition

Clinical outcomes	Patients with ARDS by either Berlin-CXR or Berlin-LUS definition (*n* = 295)	Patients with ARDS by Berlin-CXR definition (*n* = 216)	Patients with ARDS by Berlin-LUS, but not Berlin-CXR definition (*n* = 79)	*P* value^a^
28-day ventilator-free days	23 (20–25)	23 (19–25)	24 (20–25)	0.132
ICU LOS (days)	7 (5–12)	8 (5–12)	7 (4–12)	0.300
Hospital LOS (days)	19 (10–40)	18 (10–44.5)	21 (9–31)	0.712
ICU mortality, *n* (%)	59 (20.0)	42 (19.4)	17 (21.5)	0.743
Hospital mortality, *n* (%)	101 (34.2)	78 (36.1)	23 (29.1)	0.272

Values are shown as median (interquartile range) unless otherwise indicated

*ARDS* Acute respiratory distress syndrome, *CXR* chest radiography, *ICU* Intensive care unit, *LOS* Length of stay, *LUS* lung ultrasound

^a^Comparing patients who met Berlin-CXR definition versus patients who met Berlin-LUS but not Berlin-CXR definition

Using various lung ultrasound-based definitions for ARDS, the observed ICU and hospital mortality rates were similar when the lung ultrasound criterion required at least one to at least three regions of each hemithorax affected by multiple B lines (> 2 B lines per region) or consolidation on lung ultrasound (see Additional file 3: Table S3). The observed mortality rates increased when the lung ultrasound criterion for ARDS required more than three regions of each hemithorax to be affected. Across the full range of lung ultrasound criteria (from at least one to at least six regions of each hemithorax affected), the number of patients identified to have ARDS dropped drastically (from 229 to only 6 patients), resulting in sensitivity decreasing from 69% to 2%, and specificity increasing from 67% to 99% (see Additional file 4: Table S4).

## Discussion

In this retrospective study of 456 patients who required ventilatory support for hypoxemic respiratory failure, 47–50% were identified to have ARDS according to the Berlin definition using either chest radiography or lung ultrasound. The main findings were as follows: firstly, both definitions could identify similar groups of patients who had increased ICU or hospital mortality; secondly, the Berlin definition using lung ultrasound helped identify patients at higher risk of death, even if these patients did not fulfill the conventional Berlin definition using chest radiography; and thirdly, the overlap of patients using both definitions was only 65.5–69.4%.

Among 1550 patients admitted to our ICU within the study period, 216 patients met the Berlin-CXR definition for ARDS. This proportion of 13.9% was slightly higher than the 10% found in the Large Observational Study to Understand the Global Impact of Severe Acute Respiratory Failure (LUNG SAFE) [17], which may be due to our case-mix as a tertiary-level medical ICU for pneumonia and respiratory failure. Among the 216 ARDS patients identified using the Berlin-CXR definition in our study, the ventilator settings (mean positive end-expiratory pressure 7.4 cmH_2_O, mean plateau pressure 21.3 cmH_2_O) were slightly lower than those reported in the LUNG SAFE study (mean positive end-expiratory pressure 8.4 cmH_2_O, mean plateau pressure 23.2 cmH_2_O), which could be due to a greater proportion of mild ARDS in our study (39.4%) compared with the LUNG SAFE study (30.0%).

In our study, as for the Kigali Modification, we considered sonographic consolidation in addition to B lines since the former could also be a pathologic entity for ARDS [2, 8]. Additionally, we used a standard protocol for scanning six points of each hemithorax [2, 18]. Despite equivalent mortality discrimination using either the Berlin-CXR or Berlin-LUS definitions, the overlap of identified patients was only about two-thirds, and Berlin-LUS only had a sensitivity of 69% and a specificity of 67% when Berlin-CXR was taken as the diagnostic reference standard. This is plausible since the correlation of lung ultrasound and chest radiography was previously found to be moderate for the diagnosis of ARDS. In a prior pilot study covering three points of each hemithorax [9], the finding of multiple B lines bilaterally and involving at least three lung fields in total (i.e., interstitial syndrome) was only 80% sensitive and 62% specific for the diagnosis of ARDS using the Berlin definition as the reference standard. The authors explained that 25% of the study’s 77 patients were trauma patients, and such patients made lung ultrasound image acquisition and interpretation difficult.

Another reason for the limited concordance between chest radiography and lung ultrasound is that lung ultrasound could only detect abnormalities in contact with the visceral pleura, while chest radiography could detect parenchymal abnormalities away from the pleura (i.e., the sensitivity of lung ultrasound is limited by false negatives) [4]. Additionally, lung ultrasound may lack sensitivity compared with chest radiography since ARDS is generally a posterior-predominant disease and it may be difficult to scan the posterior regions of the lung due to obstruction of ultrasound by the scapula bone and the difficulty in rolling patients who are ventilated. Nonetheless, as shown in Table 3, more B lines and consolidations were detected by ultrasound in the posterior and inferior regions, rather than in the anterior and superior regions, suggesting that ultrasound is more sensitive for picking up opacities from atelectasis in the bases than chest radiography.

Chest radiography interpretation could also be falsely positive in ARDS due to the limited reliability of chest radiography, even when read by experts. In a prior study involving 21 international experts, the chance-corrected interobserver agreement was moderate (kappa 0.55) [19]. In another study involving two intensivists and a radiologist, interobserver agreement was similarly moderate (kappa 0.38–0.55) [20]. Another reason for false positive radiography could be that pleural effusion and basal pulmonary infiltrates may be indistinguishable on radiography, further limiting the apparent sensitivity of lung ultrasound (although this discordance was only present in five patients in our study). On the other hand, lung ultrasound could also be falsely positive for ARDS, thus limiting specificity. For instance, B lines could be seen in interstitial fibrosis [19], and consolidation could be seen in simple atelectasis (these same problems can be seen with chest radiography too).

While crafting the definition for ARDS, the Berlin definition workgroup primarily aimed to develop a robust and replicable definition. The workgroup secondarily supported their consensus definition using predictive validity for patient mortality. This means that, among patients with respiratory failure, the definition for ARDS should identify patients who are at higher risk of death. In their landmark paper for the Berlin definition, the workgroup found that the Berlin definition was not only able to identify patients with higher mortality but was further able to stratify patients with increasing mortality according to worsening oxygenation. We found similar results when we used the Berlin definition for our patients: the definition predicted hospital mortality, and patients with severe ARDS had worse mortality compared with patients with mild ARDS.

As for the Berlin definition, we found that modifying the chest imaging criterion—by replacing chest radiography with lung ultrasound—could also predict worse patient outcomes, with increased ICU mortality and increased ICU length-of-stay achieving statistical significance. Hospital mortality for patients also trended higher but did not achieve statistical significance, probably due to a limited sample size. As shown in Table 4, patients with ARDS by the Berlin-LUS but not the Berlin-CXR definition had almost identical outcomes to those with ARDS identified by the Berlin-CXR definition. This suggests that the former 79 patients, who would have been missed if we had used chest radiography alone, behaved like ARDS patients. Nonetheless, these 79 patients were not worse off, perhaps because our ICU applied the same ventilator strategy for both ARDS and non-ARDS patients. In the current study, for these 79 patients compared with the 216 patients with ARDS by the Berlin-CXR definition, tidal volume settings were similar (6.6 ± 2.3 versus 7.1 ± 3.2 ml/kg, *P* = 0.141) and positive end-expiratory pressure was only slightly lower (6.6 ± 2.2 versus 7.4 ± 3.1 cmH_2_O, *P* = 0.015).

Interestingly, in our study (see Table 5), patients with moderate ARDS had lower mortality compared with patients with mild ARDS (and even compared with patients without ARDS according to the Berlin-CXR definition). This could be due to classifying all patients who were on noninvasive ventilation as mild, and all patients who were not on any ventilation as no ARDS. Patients with more severe disease could then have been classified as no or mild ARDS, based on treatment modality, regardless of underlying disease severity.

**Table 5:** ICU and hospital mortality according to severity of ARDS using various definitions

Definition	Severity^a^	Number of patients	ICU mortality (%)	Hospital mortality (%)
Berlin-CXR	No ARDS	240	37 (15.4)	62 (25.8)
	Mild ARDS	85	19 (22.4)	31 (36.5)
	Moderate ARDS	62	5 (8.1)	16 (25.8)
	Severe ARDS	69	18 (26.1)	31 (44.9)
Berlin-LUS	No ARDS	227	27 (11.9)	61 (26.9)
	Mild ARDS	92	24 (26.1)	30 (32.6)
	Moderate ARDS	73	10 (13.7)	23 (31.5)
	Severe ARDS	64	18 (28.1)	26 (40.6)
Berlin-CXR or Berlin-LUS	No ARDS	161	20 (12.4)	39 (24.2)
	Mild ARDS	120	28 (23.3)	40 (33.3)
	Moderate ARDS	93	11 (11.8)	28 (30.1)
	Severe ARDS	82	20 (24.4)	33 (40.2)

*ARDS* acute respiratory distress syndrome, *CXR* chest radiography, *ICU* intensive care unit, *LUS* lung ultrasound

^a^Mild ARDS: ratio of arterial partial pressure of oxygen to inspired fraction of oxygen (P/F) 201–300 mmHg and positive end-expiratory pressure (PEEP)/continuous positive airway pressure (CPAP) ≥5 cmH_2_O using noninvasive ventilation (which may include CPAP or bi-level positive airway pressure) or invasive mechanical ventilation (IMV); moderate ARDS: P/F 101–200 mmHg and PEEP ≥ 5 cmH_2_O using IMV only; severe ARDS: P/F ≤ 100 mmHg and PEEP ≥ 5 cmH_2_O using IMV only

Based on the Kigali Modification of the Berlin definition, apart from changes to the oxygenation and positive end-expiratory pressure criteria, chest radiography could be replaced by lung ultrasound [2]. To fulfill the chest imaging criterion for bilateral opacities, the Kigali Modification proposed that at least one area of B lines and/or consolidation without associated effusion should be present in each hemithorax. We used the same threshold of at least one affected area per hemithorax in the Berlin-LUS definition and found that it performed optimally for identifying patients with ARDS, maintaining both sensitivity and specificity above 65% with respect to Berlin-CXR while maximizing the number of patients identified. As expected, making the threshold more stringent would improve specificity, but would reduce sensitivity and the number of patients identified markedly.

Given the potential for both chest radiography to miss early ARDS changes and lung ultrasound to miss pathology distant to the pleura, we propose that chest radiography and lung ultrasound could be complementary modalities for the identification of ARDS. Case identification using a combined approach (patients could fulfill either the Berlin-CXR or Berlin-LUS definitions) resulted in a 37% increase in the number of ARDS cases over the Berlin-CXR definition alone while maintaining a specificity of 67%. Identification of patients with ARDS using lung ultrasound, who would otherwise have been missed using chest radiography, could allow for appropriate disposition and treatment, and could increase the number of patients who may be recruited for research.

The main strength of our study was the availability of an established practice of performing both chest radiography and systematic lung ultrasound for patients on admission. This allowed us to perform a comparative analysis using various definitions for ARDS. Another strength is the larger number of patients in our study compared with prior published work on lung ultrasound in ARDS [11, 12].

Our study had some limitations. Firstly, we did not adjudicate the chest radiography and lung ultrasound reports that have been reported by specialist radiologists and our trained respiratory therapists, respectively. It is likely that interobserver variability within each imaging modality would exist [2] and that each imaging modality would have an imperfect correlation with a computed tomography gold standard [3, 21], but we believe our pragmatic approach would improve the generalizability of our findings in the real world. Secondly, we included only medical ICU patients, and our findings may not be applicable to surgical ICU patients (e.g., those with traumatic ARDS). Nonetheless, as our patients had no major cardiothoracic operations and had no large dressings on their chest, we could perform chest radiography and lung ultrasound for all our patients, improving the completeness of data collection. Thirdly, for routine lung ultrasound to be feasible, we scanned only six points per hemithorax and could have missed pathological findings found in between those points. Fourthly, our study involved patients from a single ICU, and validation of our findings in other ICUs would be required. Finally, the lung ultrasounds were all performed by respiratory therapists, and not by the treating physician, and this may not be easily implemented in all institutions.

Further work to be done includes testing the different definitions for ARDS in other settings and for patients with nonmedical conditions. Future studies could also test the predictive validity, feasibility, clinical benefit, and epidemiological impact of a combined chest imaging approach using both chest radiography and lung ultrasound.

## Conclusions

In conclusion, for the identification of ARDS with the Berlin definition, both chest radiography and lung ultrasound were equally related to mortality. The moderate overlap of patients when chest imaging modalities differed suggest that chest radiography and lung ultrasound should be complementary rather than used interchangeably.

## Additional files

Additional file 1: **Table S1.** Lung ultrasound reporting template. (DOCX 18 kb)

Additional file 2: **Table S2.** Treatment parameters and clinical outcomes using various definitions of acute respiratory distress syndrome. (DOCX 21 kb)

Additional file 3: **Table S3.** Comparative analysis using lung ultrasound findings for ARDS identification. (DOCX 23 kb)

Additional file 4: **Table S4.** Diagnostic accuracy using various definitions of acute respiratory distress syndrome compared with Berlin-CXR. (DOCX 22 kb)

## Abbreviations

ARDS: Acute respiratory distress syndrome
CXR: Chest radiography
ICU: Intensive care unit
LUNG SAFE: Large Observational Study to Understand the Global Impact of Severe Acute Respiratory Failure
LUS: Lung ultrasound
