# Supplementary material for: Chest radiography versus lung ultrasound for identification of acute respiratory distress syndrome: a retrospective observational study
Source: Crit Care. 2018 Aug 18;22:203. doi: 10.1186/s13054-018-2105-y (PMC6098581; doi:10.1186/s13054-018-2105-y)
Supplement: Supplementary file 1 — Table S1. Lung ultrasound reporting template. (DOCX 18 kb) [file 13054_2018_2105_MOESM1_ESM.docx]

# TABLE S1. Lung ultrasound reporting template

| Name of patient |  |
| --- | --- |
| Date of ultrasound |  |
| Time of ultrasound |  |
| Type of ultrasound | - Newly intubated patient - Spontaneous breathing trial - Other, state: |
| Right anterior lung, upper zone | - A lines, lung sliding, 0-2 B lines - A lines, no lung sliding, no B lines, positive lung pulse - A lines, no lung sliding, no B lines, no lung pulse - B lines (3 or more), separated - B lines (3 or more), coalesced - Consolidation |
| Right anterior lung, lower zone | - A lines, lung sliding, 0-2 B lines - A lines, no lung sliding, no B lines, positive lung pulse - A lines, no lung sliding, no B lines, no lung pulse - B lines (3 or more), separated - B lines (3 or more), coalesced - Consolidation |
| Right lateral lung, upper zone | - A lines, lung sliding, 0-2 B lines - A lines, no lung sliding, no B lines, positive lung pulse - A lines, no lung sliding, no B lines, no lung pulse - B lines (3 or more), separated - B lines (3 or more), coalesced - Consolidation |
| Right lateral lung, lower zone | - A lines, lung sliding, 0-2 B lines - A lines, no lung sliding, no B lines, positive lung pulse - A lines, no lung sliding, no B lines, no lung pulse - B lines (3 or more), separated - B lines (3 or more), coalesced - Consolidation |
| Right posterior lung, upper zone | - A lines, lung sliding, 0-2 B lines - A lines, no lung sliding, no B lines, positive lung pulse - A lines, no lung sliding, no B lines, no lung pulse - B lines (3 or more), separated - B lines (3 or more), coalesced - Consolidation |
| Right posterior lung, lower zone | - A lines, lung sliding, 0-2 B lines - A lines, no lung sliding, no B lines, positive lung pulse - A lines, no lung sliding, no B lines, no lung pulse - B lines (3 or more), separated - B lines (3 or more), coalesced - Consolidation |

| Left anterior lung, upper zone | - A lines, lung sliding, 0-2 B lines - A lines, no lung sliding, no B lines, positive lung pulse - A lines, no lung sliding, no B lines, no lung pulse - B lines (3 or more), separated - B lines (3 or more), coalesced - Consolidation |
| --- | --- |
| Left anterior lung, lower zone | - A lines, lung sliding, 0-2 B lines - A lines, no lung sliding, no B lines, positive lung pulse - A lines, no lung sliding, no B lines, no lung pulse - B lines (3 or more), separated - B lines (3 or more), coalesced - Consolidation |
| Left lateral lung, upper zone | - A lines, lung sliding, 0-2 B lines - A lines, no lung sliding, no B lines, positive lung pulse - A lines, no lung sliding, no B lines, no lung pulse - B lines (3 or more), separated - B lines (3 or more), coalesced - Consolidation |
| Left lateral lung, lower zone | - A lines, lung sliding, 0-2 B lines - A lines, no lung sliding, no B lines, positive lung pulse - A lines, no lung sliding, no B lines, no lung pulse - B lines (3 or more), separated - B lines (3 or more), coalesced - Consolidation |
| Left posterior lung, upper zone | - A lines, lung sliding, 0-2 B lines - A lines, no lung sliding, no B lines, positive lung pulse - A lines, no lung sliding, no B lines, no lung pulse - B lines (3 or more), separated - B lines (3 or more), coalesced - Consolidation |
| Left posterior lung, lower zone | - A lines, lung sliding, 0-2 B lines - A lines, no lung sliding, no B lines, positive lung pulse - A lines, no lung sliding, no B lines, no lung pulse - B lines (3 or more), separated - B lines (3 or more), coalesced - Consolidation |
| Other comments | - Right pleural effusion present - Right pleural effusion absent - Left pleural effusion present - Left pleural effusion absent - Right subcutaneous emphysema present - Right subcutaneous emphysema absent - Left subcutaneous emphysema present - Left subcutaneous emphysema absent |
