# Supplementary material for: Chest radiography versus lung ultrasound for identification of acute respiratory distress syndrome: a retrospective observational study
Source: Crit Care. 2018 Aug 18;22:203. doi: 10.1186/s13054-018-2105-y (PMC6098581; doi:10.1186/s13054-018-2105-y)
Supplement: Supplementary file 2 — Table S2. Treatment parameters and clinical outcomes using various definitions of acute respiratory distress syndrome. (DOCX 21 kb) [file 13054_2018_2105_MOESM2_ESM.docx]

# TABLE S2. Treatment parameters and clinical outcomes using various definitions of acute respiratory distress syndrome

| **Parameters/ clinical outcomes** | **All patients** | **Berlin-CXR** | | **Berlin-LUS** | | **Berlin-CXR or Berlin-LUS** | |
| --- | --- | --- | --- | --- | --- | --- | --- |
|  |  | **ARDS present** | **ARDS absent** | **ARDS present** | **ARDS absent** | **ARDS present** | **ARDS absent** |
| No. of patients | 456 | 216 | 240 | 229 | 227 | 295 | 161 |
| **Initial treatment parameters** |  |  |  |  |  |  |  |
| Ventilation type  Non-invasive  Invasive | 97 (21.3)  359 (78.7) | *  64 (29.6)  152 (70.4) | *  33 (13.8)  207 (86.3) | *  77 (33.6)  152 (66.4) | *  20 (8.8)  207 (91.2) | *  89 (30.2)  206 (69.8) | *  8 (5.0)  153 (95.0) |
| Initial PEEP,  mean ± SD (cmH2O) | 6.5 ± 2.6 | 7.4 ± 3.1* | 5.8 ± 1.9* | 7.5 ± 3.1* | 5.7 ± 1.8* | 7.2 ± 2.9* | 5.6 ± 1.6* |
| Initial plateau pressure^1^,  mean ± SD (cmH2O) | 19.8 ± 6.1 | 21.3 ± 6.0* | 18.7 ± 6.0* | 20.5 ± 5.8* | 19.3 ± 6.4* | 20.8 ± 5.8* | 18.4 ± 6.3* |
| Initial tidal volume/kg IBW^1^, mean ± SD (ml/kg) | 7.0 ± 2.8 | 7.1 ± 3.2 | 7.0 ± 2.4 | 6.8 ± 2.4 | 7.2 ± 3.0 | 6.9 ± 3.0 | 7.2 ± 2.4 |
| **Clinical outcomes** |  |  |  |  |  |  |  |
| 28-day ventilator-free days, median (IQR) (days) | 24 (20-25) | 23 (19-25)* | 24 (20-26)* | 24 (20-25) | 24 (19-26) | 23 (20-25)* | 24 (20-26)* |
| ICU LOS, median (IQR) (days) | 7 (4-11) | 8 (5-12)* | 6 (4-10)* | 7 (5-12)* | 6 (4-11)* | 7 (5-12)* | 6 (4-10)* |
| Hospital LOS,  median (IQR) (days) | 17 (10-39) | 18 (10-44.5) | 17 (9-33.5) | 20 (10-40) | 16 (9-38) | 19 (10-40) | 16 (9-35) |
| ICU mortality (%) | 79 (17.3) | 42 (19.4) | 37 (15.4) | 52 (22.7)* | 27 (11.9)* | 59 (20.0)* | 20 (12.4)* |
| Hospital mortality (%) | 140 (30.7) | 78 (36.1)* | 62 (25.8)* | 79 (34.5) | 61 (26.9) | 101 (34.2)* | 39 (24.2)* |

ARDS: Acute respiratory distress syndrome
IBW: Ideal body weight (kg); males = 50.0 + 0.91 (height [cm] - 152.4); females = 45.5 + 0.91 (height [cm] - 152.4)
ICU: Intensive care unit
IQR: Interquartile range
LOS: Length of stay
PEEP: Positive end-expiratory pressure
^1^ For patients who were on invasive ventilation initially only
* P<0.05. For ventilation type, a 3 x 2 Pearson chi-squared test was done for each ARDS definition
