# Supplementary material for: Chest radiography versus lung ultrasound for identification of acute respiratory distress syndrome: a retrospective observational study
Source: Crit Care. 2018 Aug 18;22:203. doi: 10.1186/s13054-018-2105-y (PMC6098581; doi:10.1186/s13054-018-2105-y)
Supplement: Supplementary file 3 — Table S3. Comparative analysis using lung ultrasound findings for ARDS identification. (DOCX 23 kb) [file 13054_2018_2105_MOESM3_ESM.docx]

# TABLE S3. Comparative analysis using lung ultrasound findings for ARDS identification

| **Patients and clinical outcomes** | **Berlin-LUS1** | **Berlin-LUS2** | **Berlin-LUS3** | **Berlin-LUS4** | **Berlin-LUS5** | **Berlin-LUS6** |
| --- | --- | --- | --- | --- | --- | --- |
| No. of patients identified to have ARDS using the respective definitions | 229 | 134 | 67 | 45 | 28 | 6 |
| 28-day ventilator-free days, median (IQR) (days) | 24 (20-25) | 23 (19-25) | 22 (17-25) | 21 (15-25) | 21 (15-24) | 21 (18-24) |
| ICU LOS, median (IQR) (days) | 7 (5-12) | 8 (5-13) | 8 (5-13) | 8 (5-14) | 8 (5-15) | 7 (6-9) |
| Hospital LOS,  median (IQR) (days) | 20 (10-40) | 20 (12-44) | 19 (12-46) | 18 (10-48) | 19 (9-55) | 52 (16-82) |
| ICU mortality (%) | 52 (22.7) | 36 (26.9) | 17 (25.4) | 15 (33.3) | 10 (35.7) | 1 (16.7) |
| Hospital mortality (%) | 79 (34.5) | 52 (38.8) | 26 (38.8) | 22 (48.9) | 16 (57.1) | 4 (66.7) |

Berlin-LUS1: Berlin Definition, replacing the imaging criterion with one or more regions of each hemi-thorax affected by multiple B lines (>2 B lines per region) or consolidation on lung ultrasound
Berlin-LUS2: Berlin Definition, replacing the imaging criterion with two or more regions of each hemi-thorax affected by multiple B lines (>2 B lines per region) or consolidation on lung ultrasound
Berlin-LUS3: Berlin Definition, replacing the imaging criterion with three or more regions of each hemi-thorax affected by multiple B lines (>2 B lines per region) or consolidation on lung ultrasound
Berlin-LUS4: Berlin Definition, replacing the imaging criterion with four or more regions of each hemi-thorax affected by multiple B lines (>2 B lines per region) or consolidation on lung ultrasound
Berlin-LUS5: Berlin Definition, replacing the imaging criterion with five or more regions of each hemi-thorax affected by multiple B lines (>2 B lines per region) or consolidation on lung ultrasound
Berlin-LUS6: Berlin Definition, replacing the imaging criterion with all six regions of each hemi-thorax affected by multiple B lines (>2 B lines per region) or consolidation on lung ultrasound
ICU: Intensive care unit
IQR: Interquartile range
LOS: Length of stay
