# Supplementary material for: Chest radiography versus lung ultrasound for identification of acute respiratory distress syndrome: a retrospective observational study
Source: Crit Care. 2018 Aug 18;22:203. doi: 10.1186/s13054-018-2105-y (PMC6098581; doi:10.1186/s13054-018-2105-y)
Supplement: Supplementary file 4 — Table S4. Diagnostic accuracy using various definitions of acute respiratory distress syndrome compared with Berlin-CXR. (DOCX 22 kb) [file 13054_2018_2105_MOESM4_ESM.docx]

# TABLE S4. Diagnostic accuracy using various definitions of acute respiratory distress syndrome, compared to Berlin-CXR

| **Definition** | **TP** | **FP** | **TN** | **FN** | **Sensitivity (95% CI)** | **Specificity (95% CI)** | **PPV**  **(95% CI)** | **NPV**  **(95% CI)** |
| --- | --- | --- | --- | --- | --- | --- | --- | --- |
| Berlin-LUS1 | 150 | 79 | 161 | 66 | 0.69  (0.63-0.76) | 0.67  (0.61-0.73) | 0.66  (0.59-0.72) | 0.71  (0.65-0.77) |
| Berlin-LUS2 | 93 | 41 | 199 | 123 | 0.43  (0.36-0.50) | 0.83  (0.76-0.87) | 0.69  (0.61-0.77) | 0.62  (0.56-0.67) |
| Berlin-LUS3 | 48 | 19 | 221 | 168 | 0.22  (0.17-0.28) | 0.92  (0.88-0.95) | 0.72  (0.59-0.82) | 0.57  (0.52-0.62) |
| Berlin-LUS4 | 34 | 11 | 229 | 182 | 0.16  (0.11-0.21) | 0.95  (0.92-0.98) | 0.76  (0.60-0.87) | 0.56  (0.51-0.61) |
| Berlin-LUS5 | 21 | 7 | 233 | 195 | 0.09  (0.06-0.14) | 0.97  (0.94-0.99) | 0.75  (0.55-0.89) | 0.54  (0.50-0.59) |
| Berlin-LUS6 | 4 | 2 | 238 | 212 | 0.02  (0.00-0.05) | 0.99  (0.97-1.00) | 0.67  (0.22-0.96) | 0.53  (0.48-0.58) |

Berlin-CXR: Berlin Definition, using chest radiography as the imaging criterion
Berlin-LUS1: Berlin Definition, replacing the imaging criterion with one or more regions of each hemi-thorax affected by multiple B lines (>2 B lines per region) or consolidation on lung ultrasound
Berlin-LUS2: Berlin Definition, replacing the imaging criterion with two or more regions of each hemi-thorax affected by multiple B lines (>2 B lines per region) or consolidation on lung ultrasound
Berlin-LUS3: Berlin Definition, replacing the imaging criterion with three or more regions of each hemi-thorax affected by multiple B lines (>2 B lines per region) or consolidation on lung ultrasound
Berlin-LUS4: Berlin Definition, replacing the imaging criterion with four or more regions of each hemi-thorax affected by multiple B lines (>2 B lines per region) or consolidation on lung ultrasound
Berlin-LUS5: Berlin Definition, replacing the imaging criterion with five or more regions of each hemi-thorax affected by multiple B lines (>2 B lines per region) or consolidation on lung ultrasound
Berlin-LUS6: Berlin Definition, replacing the imaging criterion with all six regions of each hemi-thorax affected by multiple B lines (>2 B lines per region) or consolidation on lung ultrasound
CI: Clopper-Pearson confidence interval
FN: False negative
FP: False positive
NPV: Negative predictive value
PPV: Positive predictive value
TN: True negative
TP: True positive
